# Supplementary material for: Integrating behavioral, social, and technological factors in cryptocurrency investment decisions
Source: PLoS One. 2026 Apr 15;21(4):e0332256. doi: 10.1371/journal.pone.0332256 (PMC13082585; doi:10.1371/journal.pone.0332256)
Supplement: Attachment — Submitted filename: point-by-point response_1st.docx [file pone.0332256.s003.docx]

**Financial Influencers, Impulsivity, Digital Financial Literacy, and Fintech Self-efficacy: An Integrated Model of Investment Decisions in Cryptocurrency**

I would like to express my sincere gratitude to the reviewers for their insightful and constructive comments. In the revised manuscript, all modifications made in response to the reviewers’ suggestions have been clearly marked in red for ease of reference.

| Comments | Responses |
| --- | --- |
| Review #1 |  |
| The authors employ a Structural Equation Model (SEM) to analyze the investment decision-making process of young investors in cryptocurrency markets in Vietnam. The question is relevant and interesting, and the sample used appears to be particularly noteworthy. However, three main issues should be addressed to make the analysis publication-worthy:  1. Meaning of Variables  First and most importantly, authors must clarify what their primary outcome variable means: Investment Decisions (ID). Prima facie, one would think that it refers to the decision to participate or not in cryptocurrency markets, which is what the research hypothesis development seems to imply. However, once we examine the actual questions that comprise this construct and carefully read Juwitta et al. (2022), where the questions originated, we realize that it may be risk aversion that the authors are actually measuring. This outcome variable must be made more explicit; otherwise, the entire SEM is completely misspecified. | Thanks for the helpful feedback. I have clarified the definition and meaning of the variable “Investment Decisions”. This variable reflects the level of willingness and commitment to invest in the cryptocurrency market, not “risk aversion”. The scales have been recalibrated:   - “I plan to invest in cryptocurrency”, - “I will continue to invest in cryptocurrency”, - “I strongly recommend others to invest in cryptocurrency” - “I can stand the inconvenience caused by cryptocurrency investment”.   I have also edited the “Measurement Scales” section for consistency. |
| 2. Conceptual Map  Even if the ID variable were defined correctly, the article lacks a clear conceptual map that justifies the need to test the influence of all the variables proposed in ID. The authors employ a combination of UTAUT, SET, and TPB in their measurement equation, but fail to justify why the specific mix of constructs proposed should determine ID. They just list how different theories may explain the behavior. More is needed. The authors should at least discuss how the theories used may complement each other and discuss why they should be combined.  Finally, the discussion of Impulsivity is not clear enough, either, but this is probably a result of the indeterminacy surrounding the ID variable. | I would like to thank the reviewer for his insightful comments.  In the revised version, I added a synthesis that shows the relationship between the four foundational theories including UTAUT, TPB, Self-efficacy Theory and Behavioral Finance Theory. Specifically, UTAUT explains the technological factor and social influence; TPB reflects the cognitive and intentional behavior aspects; Self-efficacy moderates the relationship between investment attitudes and behavior; while Behavioral Finance adds the emotional and impulsive factors.  In Table 1. Summary of underpinning theories, I added the Limitation of each theory in Cryptocurrency context to clarify the reason for the combination of theories  In addition, I also show the basis of combining the ideas integrated in the model, which includes the rational, social, personal ability and irrational aspects in investment decisions. |
| 3. Measurement of Variables  The definitions and names of the variables are unclear. I understand that the authors use previously published variables; however, the naming conventions and wording of the questions are not recognizable to an international audience. For example, the variable named "Investment decisions" includes questions like "You will never hang-gliding or bungee jumping in the cryptocurrency market". This wording may be influenced by Vietnamese conventions or Gen Z speech patterns, but it lacks context and therefore makes little sense to a broader audience.  This is also a problem with measuring Digital Financial Literacy. Question F4 hardly measures financial literacy. It measures the investor's confidence or overconfidence.  The attitude towards investment variable also has problems, as question AT3 refers to stocks, not cryptocurrency. | Thank you for your valuable comments.  In the revised version, I clarified the definition of each research variable (in the Research methodology section), and recalibrated the scale of Digital Financial Literacy, Investment decisions, Attitude Toward Investment (AT3), and Social media financial influencers. |
| 4. Minor issues.  - The discussion of behavioral finance is too vague and seems to equate it with the study of Impulsivity. This is not the case.  - The claim that research has "seldom integrated psychological, social, and technological factors into a unified model" is far too strong.  - The argument that FinTech facilitates transactions but also increases risk, given its importance to the hypothesis development, should be made explicit, not just dealt with in one reference. | Thanks for your input.   - I have expanded and clarified the discussion of Behavioral Finance Theory, emphasizing that it is a general theory of cognitive and psychological biases that influence financial decisions, with Impulsivity being considered as just one specific behavioral bias. - In the revised version, the sentence "seldom integrated psychological, social, and technological factors into a unified model" has been adjusted to read: “Although several studies have examined psychological, social, and technological factors separately, few have comprehensively integrated them into a unified framework.” - Add the dual role of FinTech. |
| Review #2 |  |
| 1. Title, Abstract, and Keywords  • The title is clear but overly long. It may be shortened to emphasize the central contribution (e.g., “Integrating Behavioral, Social, and Technological Factors in Cryptocurrency Investment Decisions”) | Thank you very much for your guidance, the title was revised as “Integrating Behavioral, Social, and Technological Factors in Cryptocurrency Investment Decisions”. |
| The claim that “research has seldom integrated psychological, social, and technological factors” is overstated—recent studies (2023–2025) have done so, including those cited later (e.g., Rijanto & Utami, 2024; Hassan et al., 2024). | In the revised version, the sentence "seldom integrated psychological, social, and technological factors into a unified model" has been adjusted to read: “Although several studies have examined psychological, social, and technological factors separately, few have comprehensively integrated them into a unified framework.” |
| 2. Introduction  • The introduction is detailed but too long and descriptive, reading more like a literature review than a problem statement.  • The research gap is stated in general terms (lack of integrated frameworks) but not theoretically or empirically substantiated. The authors should demonstrate how their model advances behavioral finance theory beyond existing TPB–UTAUT integrations already seen in recent work (e.g., Bouteraa, 2024; Hassan et al., 2024).  • The motivation for focusing on Vietnam is clear but underdeveloped; comparative insights or justification for cultural relevance (e.g., collectivist society, crypto regulation) would strengthen the contextual contribution. | Thank you for your comments.  In the revised version, the Introduction section has been shortened and focused on the research problem, theoretical gaps and main contributions.  The gap has been clarified with specific evidence from previous studies, and at the same time, highlighting how this study extends behavioral finance theory through the integration of impulsivity and fintech self-efficacy.  The Vietnamese context section has also been added, emphasizing the characteristics of collective culture, the influence of social networks and the incomplete legal environment. |
| 3. Theoretical Framework and Hypotheses  • The integration of four theories (UTAUT, TPB, Self-efficacy, Behavioral Finance) is ambitious but insufficiently justified. The paper lists theories rather than explaining how their constructs conceptually interlock.  • Some hypotheses are mechanical restatements of prior literature rather than theory-driven. For example:  o H5 (Impulsivity → Investment Decision) lacks novelty and empirical justification for cryptocurrency specifically.  o H8 (moderation by Fintech Self-Efficacy) should include theoretical rationale—why does self-efficacy strengthen, not weaken, the attitude–behavior link?  • Several hypotheses (H9–H11) use mediation/moderation terms interchangeably without explicit conceptual diagrams or theoretical logic for directionality. | Thank you for your valuable comments.  In the revision, the author clarified the theoretical basis for integrating the four theories, which is also presented with the limitations of each theory in the context of cryptocurrencies.  The hypotheses have been adjusted in a theoretical direction.  For H5, the author has added an argument about the specificity of impulsive behavior in the context of cryptocurrencies.  Hypothesis H8 is extended with the theoretical basis of Bandura (1977) to explain the enhancing role of fintech self-efficacy on the attitude–behavior relationship.  In addition, the mediation and moderation hypotheses (H9–H11) have been more clearly distinguished, adding an explanation of the impact arrows in the research model |
| 4. Methodology  • Measurement scales are adopted from diverse sources, but no evidence of translation process, or pilot testing is provided. Reliability is reported (Cronbach’s alpha), yet discriminant validity is only briefly assessed (HTMT table) but the discussion seems as Pearson correlation discussion.  • The study uses PLS-SEM, which is suitable for exploratory models; however, justification for choosing PLS over covariance-based SEM is not provided. The authors should explain why PLS was used (e.g., non-normal data, model complexity).  • Common method bias (CMB) is likely, as all data are self-reported. The paper should apply Harman’s single-factor test or VIF statistics to mitigate this concern.  • Multivariate normality is not assessed. | Thanks for your comment.  The author has revised and explained in detail the discriminant validity test, based on the HTMT index as recommended by Henseler et al. (2015).  The author also tested the common method variance (CMB) using Harman’s single-factor test and VIF, and the results showed that there were no serious problems with CMB.  Multivariate normality test was also added to ensure the reliability of the data before conducting the structural model analysis. |
| 5. Results and Interpretation  • All eleven hypotheses are supported, which is statistically improbable and suggests potential model overfitting or confirmation bias.  • confidence intervals should be reported  • The f² and Q² table is mislabelled (“Bảng” indicates incomplete editing). Effect sizes are inconsistently interpreted—particularly for the mediated relationship, are described without backing.  • The mediation results (H9–H11) are not shown through indirect path coefficients or confidence intervals; these are essential for transparency.  • The interpretation remains descriptive, repeating numerical findings without deeper behavioral or cultural interpretation (e.g., why impulsivity still exerts a positive effect in Vietnam). | Thank you for your comments.  Due to the need to modify some scales, the survey results with these new scales have a statistically insignificant hypothesis 7.  The author added confidence intervals for each relationship, and clarified the indirect path coefficients and the results of the mediation test (H9–H11).  The f² and Q² tables have been labeled and interpreted consistently according to Cohen's threshold, and additional content to evaluate the results.  The results and interpretation section has been expanded to further explain the behavior and cultural context of Vietnamese investors, especially explaining why Impulsivity still has a positive impact in the high-risk crypto investment environment where psychological factors and social influences play a strong role. |
| 6. Discussion and Contributions  • The discussion section repeats the results rather than linking them to broader behavioral finance or fintech literature.  • The claimed theoretical contributions (extending TPB, UTAUT, self-efficacy) are not demonstrated empirically; instead, they remain declarative.  • Practical implications are extensive but lack prioritization or feasibility analysis (e.g., “regulatory oversight” and “public education campaigns” are policy-level actions requiring evidence-based design). | Thank you for your valuable comments.  The author has adjusted the discussion content to be more closely linked to the theoretical foundation of behavioral finance and technology acceptance, clarifying how psychological, social and technological factors interact in cryptocurrency investment behavior.  Theoretical contributions are concretized through the mediating role of investment attitudes and the moderating role of FinTech self-efficacy.  In terms of practical implications, the study has added the priority and feasibility section, focusing on specific measures such as financial education programs, FinTech training instead of just general policy recommendations. |
| 7. Limitations and Future Research  • The limitations are appropriately listed (self-report, cross-sectional, young sample), but no mitigation strategies are suggested. Including remedies (e.g., longitudinal or multi-source data) would enhance credibility.  • Future research directions should identify specific constructs or moderating variables (e.g., financial regulation awareness, perceived risk, or crypto trust) rather than restating standard methodological fixes. | Thank you for your comments.  The study added measures to overcome the limitations, such as proposing to incorporate real life behavioral data, expanding the research sample, and applying a longitudinal research design to increase reliability and generalizability. I  n addition, specific future research directions such as financial regulatory awareness, risk perception, and trust in cryptocurrency platforms are proposed to strengthen the theoretical and practical value. |
| 8. Writing Quality and Formatting  • The manuscript is mostly well-written but shows signs of AI-assisted phrasing—overly polished transitions (“Taken together,” “This study enriches,” etc.) and uniform sentence rhythm.  • Some references lack full details or formatting consistency.  • Figures and tables are not numbered or cross-referenced properly (e.g., “Fig 1” lacks caption clarity) | Thank you for your comments.  The entire article has been revised to ensure more natural and diverse sentence structure and transitions.  The list of references has been reviewed and revised.  In addition, tables and figures have been numbered, with additional titles and clear references in the article content. |
| 9. Ethical and Data Transparency  • The data availability statement (“available on request”) conflicts with PLOS ONE’s open-data policy. Data should be deposited in a public repository with anonymized files unless privacy law prohibits it.  • The AI acknowledgment is transparent but should include the exact purpose (“used for grammar improvement only”). | Thanks for your guidance.  The research data will be stored in accordance with PLOS ONE policy, with the files anonymized.  The AI acknowledgment has been edited to clarify that ChatGPT was used only to improve grammar during the manuscript editing process |
