# Supplementary material for: Integrating behavioral, social, and technological factors in cryptocurrency investment decisions
Source: PLoS One. 2026 Apr 15;21(4):e0332256. doi: 10.1371/journal.pone.0332256 (PMC13082585; doi:10.1371/journal.pone.0332256)
Supplement: Attachment — Submitted filename: point-by-point response_2r detail.docx [file pone.0332256.s004.docx]

**Financial Influencers, Impulsivity, Digital Financial Literacy, and Fintech Self-efficacy: An Integrated Model of Investment Decisions in Cryptocurrency**

I would like to express my sincere gratitude to the reviewers for their insightful and constructive comments. In the revised manuscript, all modifications made in response to the reviewers’ suggestions.

**Review #3**

**Review Comments to the Author**

The current policy implications are too descriptive and broad. They primarily emphasize the significance of education, regulation, and protections based on platforms, but they lack the comprehensive cross-sector framework needed to manage a complicated digital-financial ecosystem. To enhance this section, the authors ought to integrate perspectives from three essential works—Steif’s Public Policy Analytics, Weimer & Vining’s Policy Analysis: Concepts and Practice, and Gohwong’s (2021) comparative analysis of crypto governance. These sources aid in defining a more transparent cross-sector rationale that links regulators, banks, fintech companies, investors, universities, and digital platforms, allowing the policy implications to progress from broad suggestions to a consistent, multi-actor governance framework.

The existing policy ramifications continue to be too broad and complicated. The proposed policies represent extensive technocratic overreach grounded in contemporary policy models. The need for digital-financial education using evidence-driven targeting and behavioral insights is patronizing, intending to manipulate investor behavior instead of fostering independent decision-making. This paternalism encompasses universities, which are anticipated to mandate simulation-based training, as well as banks and fintech companies, which are required to transform their apps into digital guardians with obligatory risk assessments and expense monitoring. Additionally, the drive for adaptive oversight in monitoring influencers is merely a surveillance directive, and the government's ultimate effort to reshape the national narrative is a cynical endeavor to substitute authentic market sentiment with government-sanctioned data-driven reasoning.

References

Gohwong, S. (2021). Comparative non-government crypto policy between Thailand and Argentina: A lesson from different public policy policy. Journal of Government and Politics, 12(3), 367–392.

Steif, K. (2022). Public policy analytics: Code and context for data science in government. CRC Press.

Weimer, D. L., & Vining, A. R. (2017). Policy analysis: Concepts and practice (6th ed.). Routledge.

Note: Suggested improvements for completeness (optional if overly burdensome)

**Responses**

Based on the suggestions, the authors revised the policy implications, specifically as follows (from line 595 to line 616):

- The policy implications section has been adjusted to directly respond to criticisms of technocratic intervention and paternalism in previous proposals.
- The revision removes mandatory measures and avoids behavioral guidance, instead emphasizing voluntariness, transparency, and respect for investor autonomy.
- Digital financial education is presented as a support tool, not a mechanism for behavioral manipulation.
  The role of universities, banks, and fintech companies is shifted from “guardians” to optional providers, leaving users to decide their own level of participation.
- Influencer supervision is reinterpreted as limited and proportionate, avoiding the perception of excessive supervision.

**Review #4**

**Review Comments to the Author**

Digital Financial Literacy (DFL) Measurement

The revision of the Digital Financial Literacy scale is appreciated. However, item FL4 (“I feel that my knowledge and expertise will enable me to outperform the market”) does not measure digital literacy. Instead, it reflects confidence or overconfidence, which is conceptually distinct from literacy. Replacing or rewording this item to focus on knowledge or ability. For example, the ability to evaluate platform legitimacy or digital security would improve construct validity.

**Responses**

Item FL4 has been revised to: “I can use my knowledge to evaluate the legitimacy, risks, and security features of cryptocurrency platforms before making investment decisions” (line 433).

**Review Comments to the Author**

Behavioral Finance Framework

The discussion of Behavioral Finance is still somewhat narrow and remains framed primarily around impulsivity. Behavioral Finance encompasses a broader set of well-established cognitive and emotional biases including overconfidence, herding, anchoring, and loss aversion which should be briefly acknowledged to provide a more complete theoretical grounding.

**Responses**

In the revised version, the Behavioral Finance section has been expanded to more fully reflect cognitive and emotional biases. The author has added and explained important biases such as overconfidence, herding, anchoring, and loss aversion, instead of focusing only on impulsivity. Impulsivity is retained as a specific bias in the research context (from line 120 to line 129).

**Review Comments to the Author**

FinTech-Related Risk Mechanisms

While the manuscript refers to FinTech’s role in facilitating cryptocurrency investment and increasing risk exposure, the explanation of the underlying mechanism requires greater detail. Expanding this section to describe how digital platforms reduce friction, enable rapid decision-making, and amplify risk-taking would strengthen the argument.

**Responses**

The author has revised and expanded this section according to reviewer comments, explaining more clearly the mechanism of FinTech digital platforms that reduce transaction friction, support quick decision-making, and increase investors' risk tolerance (from line 301 to line 308).

**Review Comments to the Author**

Impulsivity Justification

Although the definitions have been clarified, the rationale for including impulsivity as a predictor remains somewhat limited. Consider strengthening this section with references to behavioral mechanisms (e.g., sensation seeking, delay discounting) or contextual factors that make impulsivity particularly relevant to crypto investment behavior.

**Responses**

The author revised the theory of impulsivity according to the reviewer’s comments. Behavioral mechanisms such as sensation seeking and delay discounting were added. The revision also emphasizes specific contextual factors of the cryptocurrency market. These include the FOMO effect and the desire for quick profits (from line 145 to line 154).

**Review Comments to the Author**

Item Wording and Clarity

A few measurement items still include metaphorical or culturally specific phrasing that may introduce interpretive ambiguity. Ensuring that all items are phrased in clear, direct, and culturally neutral language will improve response reliability. For example, bungee jumping and thrilling ride.

**Responses**

The author has reviewed the scales and adjusted them according to comments (line 433).

**Review Comments to the Author**

Writing Style and Flow

Certain sections exhibit uniform sentence rhythm and transition patterns that give the appearance of AI-assisted phrasing. A light manual edit to vary sentence structure and transitions would enhance readability.

**Responses**

The author has reviewed and edited the entire article.
