# Supplementary material for: Integrating behavioral, social, and technological factors in cryptocurrency investment decisions
Source: PLoS One. 2026 Apr 15;21(4):e0332256. doi: 10.1371/journal.pone.0332256 (PMC13082585; doi:10.1371/journal.pone.0332256)
Supplement: Attachment — Submitted filename: Response to Reviewers_3r.docx [file pone.0332256.s005.docx]

**Financial Influencers, Impulsivity, Digital Financial Literacy, and Fintech Self-efficacy: An Integrated Model of Investment Decisions in Cryptocurrency**

I would like to express my sincere gratitude to the reviewers for their insightful and constructive comments. In the revised manuscript, all modifications made in response to the reviewers’ suggestions.

**Review #5**

**Review Comments to the Author**

1. Table 5 contains internal inconsistencies. For H2 and H8, statistically significant p-values (p < 0.05) are reported alongside 95% confidence intervals that include zero (e.g., H8: [−0.062, 0.143]). Under standard two-sided inference, these statements are not jointly compatible and suggest either a transcription/labeling problem (e.g., the interval is not the stated CI) or an error in reporting.

**Responses**

Thank you for highlighting this issue. The author rechecked the bootstrapping output and found that the reported 95% confidence intervals for H2 and H8 in Table 5 contained transcription errors. The confidence interval values have now been corrected (Table 5).

**Review #4**

**Review Comments to the Author**

In addition, H4 and H5 display identical point estimates and t-statistics (β = 0.476, t = 11.031), which raises the possibility of a mistake. The authors should conduct a rigorous audit to ensure the accuracy, validity of the reported statistical results.

**Responses**

Thank you for pointing this out. The author audited the structural model results and identified a transcription error in Table 5, where the coefficients for H4 and H5 were inadvertently duplicated. The table has now been corrected (Table 5).

**Review Comments to the Author**

The Abstract is inconsistent with H1. It states that digital financial literacy reduces impulsivity, whereas Table 5 reports a strong positive effect (β = 0.729, p < 0.001). The current explanation is difficult to reconcile with items such as acting “on the spur of the moment” or “without deliberation,” which capture rashness rather than informed decisiveness. The authors should provide a theory-consistent mechanism (e.g., overconfidence, illusion of control, or social-media-driven perceived literacy) and distinguish rapid informed decisions from affect-driven impulsivity.

**Responses**

Thank you for this insightful comment. The author revised the Abstract (Line 14-25) and Discussion (line 425-435) to ensure consistency with H1 and the empirical results reported in Table 5. The revised manuscript no longer states that digital financial literacy reduces impulsivity. Instead, it clarifies that higher digital financial literacy is positively associated with impulsivity related investment behavior in the cryptocurrency context

The author also explicitly distinguish rapid informed decision making from affect driven impulsivity by emphasizing that impulsivity is measured through spontaneous and minimally deliberated actions, which capture emotional rashness rather than informed decisiveness. Drawing on behavioral finance theory, this positive relationship is explained through overconfidence and illusion of control, reinforced by perceived digital competence and social media exposure in highly volatile markets.

**Review Comments to the Author**

The policy implications should reflect H1. If higher digital financial literacy predicts higher impulsivity, recommendations focused on expanding technical literacy may be incomplete and could exacerbate impulsive trading without complementary behavioral training. The implications should explicitly emphasize behavioral discipline and emotional regulation alongside technical skills.

**Responses**

Thank you for instruction. The author has revised the Policy Implications, emphasizing the integration of behavioral discipline and emotional regulation alongside digital financial skills to mitigate impulsive trading and promote sustainable investment behavior (line 527-537; line 553-560).

**Review Comments to the Author**

H8 is interpreted inconsistently with its sign. The interaction coefficient is negative (β = −0.079), yet the text claims a strengthening effect. A negative interaction implies attenuation. Subject to corrected reporting, the authors should revise the interpretation and state the practical meaning of the negative moderation.

**Responses**

Thank you for noting this issue. The interaction coefficient for H8 was incorrectly reported during result rewriting and has been corrected to a positive value (β = 0.079) in the revised manuscript. The interpretation has been revised accordingly to reflect a small positive moderating effect and its practical meaning (line 404 – 410).

**Review #6**

Minor language and formatting issues are minimal and do not affect the scientific quality of the work. Overall, the manuscript is clearly written, coherent, and suitable for publication in its current form.

**Responses**

Thank you very much for this encouraging comment. The author has continued to review the manuscript and made minor language and formatting adjustments (all lines)
