# Supplementary material for: Integrating behavioral, social, and technological factors in cryptocurrency investment decisions
Source: PLoS One. 2026 Apr 15;21(4):e0332256. doi: 10.1371/journal.pone.0332256 (PMC13082585; doi:10.1371/journal.pone.0332256)
Supplement: Attachment — Submitted filename: Response to Reviewers_4r.docx [file pone.0332256.s006.docx]

**Financial Influencers, Impulsivity, Digital Financial Literacy, and Fintech Self-efficacy: An Integrated Model of Investment Decisions in Cryptocurrency**

I would like to express my sincere gratitude to the reviewers for their insightful and constructive comments. In the revised manuscript, all modifications made in response to the reviewers’ suggestions.

**Review #5**

**Review Comments to the Author**

1. The author has addressed all the comments, but the authenticity of the empirical results remains a cause for concern. Specifically, the coefficient for H8 was inverted from negative to positive without any corresponding change in the t-value or p-value, which is statistically implausible. Furthermore, regarding H5, while the path coefficient and t-value were updated, the confidence interval remains unchanged, suggesting a lack of rigorous re-analysis. These discrepancies cast significant doubt on the authenticity and reliability of the experimental results. Therefore, I recommend that the manuscript not be accepted until the authors provide the original software output logs (e.g., SmartPLS report) to substantiate the reported findings.

**Responses**

Thank you for your guidance. In the revised version, the author re-estimated the full PLS-SEM model, using one bootstrapping procedure with 5,000 resamples, and all results reported in Table 5. The moderating effect in H8 is now negative and statistically significant (β = –0.082, t = 2.140, p = 0.032; CI = [–0.154; –0.001]), and the sign and test statistics are fully consistent. For H5, the confidence interval was recomputed after re-running the model and now matches the updated coefficient and t-value (β = 0.163, t = 2.212, p = 0.027; CI = [0.017; 0.306]). All reported values are taken directly from the SmartPLS output.

**Review #7**

**Review Comments to the Author**

I have carefully reviewed the revised version of the manuscript, and I have three main comments that must be discussed before its publication: While they are minor, they are essential.

1. First, financial literacy teaches us rational investing, motivated by at least three main analyses: fundamental analysis, cyclical effects, and technical analysis. However, the crypto market is dominated by retail investors and weak fundamentals. Even if they do not exhibit specific cyclical effects that can be exploited using technical or other analysis. Thus, please explain this mechanism in a clearer fashion: How financial literacy motivates investing in cryptos. Perhaps some literature support comes from Ali et al. (2025). https://doi.org/10.1186/s40854-024-00686-4.

**Responses**

Thank you for this comment. The author revised the manuscript to clarify that, in cryptocurrency markets, financial literacy does not motivate investment through traditional fundamental, cyclical, or technical analysis. Instead, it supports informed participation by improving investors’ understanding of market volatility, risk exposure, and the practical use of trading platforms, as well as their ability to manage risk through diversification and hedging. The theoretical explanation has been adjusted accordingly, and recent literature has been used to support this revised mechanism.

**Review Comments to the Author**

Second, beyond crypto investing, the authors should discuss investing preferences in the Asian markets, as they have studied the Vietnamese market. In this respect, Ülkü et al. (2023) offer a comprehensive understanding of investing habitat I the Asian markets. It will help us to understand the financial decisions made by the investors, specifically retail and foreign investors, which may help the authors to enhance the clarity and generalizability of this study. https://doi.org/10.1016/j.pacfin.2023.102044.

Once these two aspects are discussed, the manuscript can be accepted as it is in good shape after three rounds of revision. Overall, it's an interesting paper and I recommend its publication.

**Responses**

Thank you for this suggestion. The author added a brief discussion in the Discussion section to situate the Vietnamese cryptocurrency market within the broader context of Asian financial markets. Specifically, the discussion highlights that retail investors dominate trading activity, tend to trade more actively during periods of uncertainty, and behave differently from foreign institutional investors, based on evidence from Asian markets.
