# Supplementary material for: Integrating behavioral, social, and technological factors in cryptocurrency investment decisions
Source: PLoS One. 2026 Apr 15;21(4):e0332256. doi: 10.1371/journal.pone.0332256 (PMC13082585; doi:10.1371/journal.pone.0332256)
Supplement: Attachment — Submitted filename: Response to Reviewers_5r.docx [file pone.0332256.s007.docx]

**Financial Influencers, Impulsivity, Digital Financial Literacy, and Fintech Self-efficacy: An Integrated Model of Investment Decisions in Cryptocurrency**

I would like to express my sincere gratitude to the reviewers for their insightful and constructive comments. In the revised manuscript, all modifications made in response to the reviewers’ suggestions.

**Review #1**

**Review Comments to the Author**

1. I share a previous referee concerns about the authenticity of the empirical results. The discrepancies previously found cast significant doubt on the authenticity and reliability of the experimental results. The authors have fixed them but not provided output logs as requested by a previous referee. Therefore, I also recommend that the manuscript not be accepted until the authors provide the original software output logs (e.g., SmartPLS report) to substantiate the reported findings. If they do, my recommendation would be to accept, as the paper is now otherwise ready.

**Responses**

Thank you for raising this concern. I have provided three original SmartPLS output files as supplementary materials. These files include the full measurement model results, structural model results, and bootstrapping outputs.

In the final revision, I rechecked all coefficients, p-values, confidence intervals, and model indicators (R², f², Q²) against the original software reports. A few minor corrections were made to ensure that the numbers and interpretations in the manuscript match the SmartPLS outputs exactly. These revisions relate only to presentation and wording. There were no changes to the dataset, the model specification, or the estimation procedure. I sincerely apologize for any confusion these inconsistencies may have caused and respectfully ask for your understanding regarding these final adjustments. The revisions were made solely to improve reporting accuracy and ensure full consistency between the manuscript and the original analytical results.
